# Relationships between Kováts Retention Indices and Molecular Descriptors of 1-(2-Hydroxy)-3-Arylpropane-1,3-Diones

**DOI:** 10.1100/tsw.2001.399

**Published:** 2001-12-14

**Authors:** Gustavo P. Romanelli, Jorge L. Jios, Juan C. Autino, Lázaro F.R. Cafferata, Eduardo A. Castro

**Affiliations:** ^1^ LADECOR, Departamento de Química, Facultad de Ciencias Exactas, Universidad Nacional de La Plata, Calles 47 y 115, La Plata 1900, Argentina; ^2^ CEQUINOR, Departamento de Química, Facultad de Ciencias Exactas, Universidad Nacional de La Plata, C.C. 962, La Plata 1900, Argentina

**Keywords:** β-diketones, QSRR theory, gas chromatography, retention index, molecular descriptors

## Abstract

Experimental and theoretical results for retention index of a set of 20 beta-diketones are given. The quantitative structure-chromatographic retention relationships (QSRR) theory is employed and six molecular descriptors are chosen to compute the fitting polynomials. Multiple regression analysis yields satisfactory results when one resorts to several variables equations, instead of computing just one-variable formulae. Average absolute deviations from experimental results are rather low, which seems to point out the suitability of the present approach.

## INTRODUCTION

1-(2-Hydroxyphenyl)-3-arylpropane-1,3-diones are the last intermediate in the Baker–Venkataraman synthesis of 2-arylchromones[1]. Standard literature registers fungicidal[2,3], bactericidal[4,5], and antitumoral[6,7] properties for some of them. The chemistry of 1,3-dicarbonyl compounds and their tautomeric forms[8,9] have been the subject of constant interest. NMR, IR[10,11,12,13,14,15,16,17,18], and mass (MS)[19,20,21] spectroscopy as well as X-ray diffraction determination[22], *ab initio*[9], and semiempirical molecular orbital calculations[23,24] have been applied to the study of the keto-enol equilibrium, the electronic and geometrical structures of both keto and enol forms, and the intramolecular OH···O hydrogen bond formed by the enol tautomer.

In a previous work we studied the tautomerism in some 1-(2-hydroxyphenyl)-3-α- and -β-naphthylpropane-1,3-diones in solution, by means of $^{1}H$, $^{13}C$ [25,26,27], and $^{17}O$[28] NMR spectroscopy, and also with single crystal X-ray diffraction and IR spectroscopy[29].

The purpose of this work is to extend the set of compounds, including other aryl and alkyl groups at the 3-position, as part of a more general study on the subject to evaluate the ability of the molecular descriptors method for predicting the Kováts retention indices (RIs).

Many quantitative structure-chromatographic retention relationships (QSRR) have shown that the chromatographic behavior can be predicted from molecular structure, which gives information about the different molecular properties that may participate in the interaction between molecules and the stationary phase in gas chromatography(GC) [30,31,32,33,34]. The RI concept, first proposed by E. Kóvats[35], has turned out to be a very useful tool for the presentation and interpretation of chromatographic data. The main advantage of RIs is the possibility of their precalculations by numerous methods[36] for comparison with experimental data from chromatographic and/or chromato-spectral methods of organic compound identifications.

The theoretical calculation of RIs is very important for the formation of GC databases[37] because the number of experimentally measured available values is not as large as the number of standard MS[38]. The key role in the realization of this possibility is the capability to attain precise RI values, so that the search for new methods of predicting RIs with a rather high degree of accuracy and suitable data quality control is very topical at present.

The aim of this work is to report new experimental values and theoretical calculations of Kóvats RIs for a set of 20 1-(2-hydroxyphenyl)-3-arylpropane-1,3-diones to test the possibility for an efficient and reliable way to obtain gas chromatographic data.

The paper is organized as follows: the next section deals with the experimental details and after that we present the theoretical framework of this study. Later on we present the calculation procedure and the numerical data. Finally, we discuss the results and analyze some of the implying derivations of this work, as well as some of the possible further extensions.

## EXPERIMENTAL DETAILS

The molecules chosen for the present study have the general form given in Fig. 1.

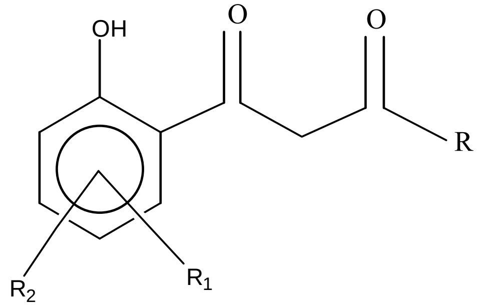

**FIGURE 1**

The complete set of 20 molecules is given in Table 1.

The synthesis of all 1-(2-hydroxyphenyl)-3-α- and -β-naphthylpropane-1,3-diones has been reported elsewhere[27,28]. The other compounds were obtained following the same preparation procedure. Retention times were obtained with a 5890 model Series II Plus Hewlett-Packard gas chromatograph equipped with a HP 5MS silica fused capillary column (5% cross-linked phenylmethylsilicone) 30-m length, 0.25-mm i.d., and 0.25-μm film thickness and coupled to an

**TABLE 1**
**1-3 Propanediones Molecular Set**

| Molecule* | R | $R_1$ | $R_2$ |
|---|---|---|---|
| **1** | α-Naphthyl | H | H |
| **2** | α-Naphthyl | 5-F | H |
| **3** | α-Naphthyl | 5-Cl | H |
| **4** | α-Naphthyl | 5-Br | H |
| **5** | α-Naphthyl | 5-Methyl | H |
| **6** | α-Naphthyl | 5-Methoxy | H |
| **7** | α-Naphthyl | 3-Methoxy | 5-Methoxy |
| **8** | α-Naphthyl | 4-Cl | H |
| **9** | β-Naphthyl | H | H |
| **10** | β-Naphthyl | 5-F | H |
| **11** | β-Naphthyl | 5-Cl | H |
| **12** | β-Naphthyl | 5-Br | H |
| **13** | β-Naphthyl | 5-Methyl | H |
| **14** | β-Naphthyl | 5-Methoxy | H |
| **15** | β-Naphthyl | 4-Cl | H |
| **16** | Phenyl | H | H |
| **17** | Phenyl | 4-Cl | H |
| **18** | 3-Pyridil | H | H |
| **19** | 3-Pyridil | 4-Cl | H |
| **20** | 3-Pyridil | 4-Br | H |

* See Figure 1.

HP 5972 A model mass selective detector. Samples were dissolved in chloroform and 2 μl of 0.01 M solutions were introduced in the injection port at 250°C (split mode) on $N_2$ carrier (constant pressure: 12.0 Psi). The oven column was maintained at 250°C as isothermal temperature program. In order to obtain the adjusted retention indices, the dead time was determined by injection of nitrogen gas.

## THEORETICAL FRAMEWORK

QSRR are some of the most extensively studied expressions of linear free-energy relationships (LFER). These are statistically derived relationships between the structure of solutes and their chromatographic retention[36]. Using QSRR, the chromatographic column can be considered as a "free-energy transducer," translating the existing differences in chemical potentials of solutes resulting from differences in their structures to chromatographic RIs. If statistically significant QSRR are derived and if these equations approximate the experimental retention data for a structurally representative set of model solutes, it is possible to define the dominant factors that determine the interactions of solute molecules with the chemical entities forming the chromatographic system[39]. It means that QSRR analysis can provide an insight into the molecular mechanism of chromatographic retention in a given chromatographic system[40].

One can distinguish two central approaches to QSRR analysis. One approach employs—as independent variables in QSRR equations—the structural descriptors provided solely by the computational chemistry. With good QSRR equations with such descriptors, one can predict retention for any given structural formula. Besides, it is also possible to assign physical meaning to the more commonly used theoretical descriptors. In turn, this procedure facilitates interpretations of the mechanism of separation operating in a given chromatographic system[41].

Another approach to QSRR employs the LFER-based empirical solute parameters based on spectroscopic complexation, and dissolution scales.

In this work we have resorted to the first approximation, choosing a set of well-known molecular descriptors to predict RIs. Quantum-chemical methods and molecular modeling techniques enable the definition of a large number of molecular and local quantities characterizing the reactivity, shape, and binding properties of complete molecules as well as of molecular fragments and substitutes. Because of the large well-defined physical information content encoded in many theoretical descriptors, their use in the design of a training set in QSRR studies present two main advantages:

1. The compounds and their various fragments and substitutes can be characterized on the basis of their molecular structure only, and
2. The proposed mechanism of action can be directly accounted for in terms of the chemical reactivity of the compounds under study[42]. Consequently, the derived QSRR model will include information regarding the nature of the intermolecular forces involved in determining the physical property of the compounds in question.

The molecular descriptors chosen for computing RIs of the beta-diketones through the employment of QSRR relate as directly as possible to the key physical chemistry property studied here. They are Van der Waals-surface-bounded molecular volume (V), the log of the octanol-water partition coefficient (log p), molecular polarizability (α), solvent-accessible surface bounded molecular volume (SAG), molar refractivity (RM), and molecular mass (M). In a previous paper Katritzky et al.[43] have pointed out that charged partial surface area descriptors have been successfully combined with topological and geometrical descriptors to predict GC RIs of substituted pyrazines, polycyclic aromatic compounds, stimulants, and narcotic and anabolic steroids. The satisfactory results found in this and other similar studies encouraged us to continue with the use of such descriptors within the realm of the QSRR to analyze this GC system.

## CALCULATION METHOD

Calculation of log p is carried out using atomic parameters derived by Ghose and coworkers[44]. Computation of molar refractivity was made via the same method as log p. Ghose and Crippen[45] have presented atomic contributions to the refractivity. The SAG and V calculations are based on a grid method derived by Bodor and coworkers[46], using the atomic radii of Gavezotti[47]. The polarizability was estimated from an additivity scheme given by Miller[48], where different increments are associated with different atom types.

The six quantum-chemical descriptors were computed with the aid of the software ChemPlus[49] and the calculations were run in a Pentium PC with 1 GHz. We have made a complete regression analysis resorting to linear, quadratic, and cubic relationships in several variables and calculations were carried out by means of the Mathematica® software[50,51].

When predicting physical chemistry properties, it is important to make full use of the intrinsic advantages of the regression formulae. In fact, we previously verified that a satisfactory improvement of these relationships can be obtained via the simple resort of employing higher-order fitting polynomials as well as by choosing noninteger powers for the independent variables[52,53,54,55,56]. Nonlinear models may be fitted to data sets by the inclusion of functions of physical chemistry parameters in a linear regression model or by use of nonlinear fitting models.

Construction of linear regression models containing nonlinear terms is most often prompted when the data are clearly not well fitted by a linear model. A very common example in the field of QSRR involves nonlinear relationships with the hydrophobic descriptors, such as log p[57].

Nonlinear dependency of molecular properties on this parameter became apparent early in the development of the QSRR model and a first approach to the solution of these drawbacks involved fitting a parabola in log p[58]. Whatever the cause of such relationships, it is clear that nonlinear functions are required in order to model the physical chemistry data. An interesting feature in the employment of nonlinear functions is that it is possible to calculate an optimum value for the physical chemistry property under consideration[59,60].

## RESULTS AND DISCUSSION

The results for the calculation of the 6 molecular descriptors corresponding to the 20 molecules given in Table 1 are displayed in Table 2.

The first set of fitting equations are those associated with the prediction of RIs vs. just one independent variable. The results are not really significant, as can be seen in Table 3 where we present the regression coefficients for the lineal, quadratic, and cubic equations, respectively.

Regression coefficients are rather poor and in some cases (i.e., log p) they are definitively unacceptable. The calculation of higher order one-variable fitting polynomials does not improve remarkably the results arising from the linear equations, save for the variables α and M.

When one resorts to several variables equations the results are quite satisfactory. In fact, as can be seen in the following typical equations, statistical data are acceptable. Here we report just some representative equations, and complete results are available upon request to E.A.C. at the above address.

$$RI/1000 = -1.7450 + 6.9 \times 10^{-3}\ SAG + 3.3 \times 10^{-3}\ M \quad (1)$$
$$n = 20,\ r = 0.9817,\ EV = 0.004164$$

$$RI/1000 = -1.0542 + 3.3 \times 10^{-3}\ V + 3.5 \times 10^{-3}\ M \quad (2)$$
$$n = 20,\ r = 0.9790,\ EV = 0.005353$$

$$RI/1000 = -0.4614 + 2.54 \times 10^{-2}\ RM + 3.2 \times 10^{-3}\ M \quad (3)$$
$$n = 20,\ r = 0.9711,\ EV = 0.007320$$

$$RI/1000 = -1.9018 + 7.3 \times 10^{-3}\ SAG - 2.64 \times 10^{-2}\ \log p + 3.5 \times 10^{-3}\ M \quad (4)$$
$$n = 20,\ r = 0.9839,\ EV = 0.004378$$

$$RI/1000 = 16.2938 - 0.0889\ SAG + 0.0001\ S^2 - 0.2343\ \log p + 0.0559\ (\log p)^2 + 0.04301\ M- - 0.0001\ M^2$$
$$n = 20,\ r = 0.9942,\ EV = 0.001923 \quad (5)$$

$$R/1000 = -1.7056 + 6.2 \times 10^{-3}\ SAG - 3.79 \times 10^{-2}\ \log p + 1.28 \times 10^{-2}\ \alpha + 3.4 \times 10^{-3}\ M$$
$$n = 20,\ r = 0.9851,\ EV = 0.004317 \quad (6)$$

where n is the number of molecules, r is the regression coefficient, and EV is the estimated variance.

In Table 4 we display some theoretical results applying several variable equations together with the experimentally determined RIs. The analysis of statistical results associated with Eq. 1 through 6 and the agreement between theoretical and experimental results shows that satisfactory predictions can be obtained when one appeals to several variable fitting polynomials.

**TABLE 2**
**Molecular Descriptors Corresponding to the Molecular Set**

| Molecule(*) | SAG | V | Log p | RM | P | M |
|---|---|---|---|---|---|---|
| **1** | 490.22 | 824.90 | 3.37 | 86.63 | 34.04 | 290.32 |
| **2** | 496.10 | 833.73 | 3.51 | 86.84 | 33.95 | 308.31 |
| **3** | 507.43 | 856.98 | 3.89 | 91.43 | 35.97 | 324.76 |
| **4** | 520.51 | 880.78 | 4.16 | 94.25 | 36.66 | 369.21 |
| **5** | 519.12 | 876.70 | 3.84 | 91.67 | 35.87 | 304.35 |
| **6** | 532.31 | 900.68 | 3.12 | 93.09 | 36.51 | 320.34 |
| **7** | 549.00 | 943.53 | 2.86 | 99.59 | 38.98 | 350.37 |
| **8** | 513.08 | 867.00 | 3.89 | 91.47 | 35.97 | 324.76 |
| **9** | 499.16 | 835.31 | 3.37 | 86.63 | 34.04 | 290.32 |
| **10** | 504.42 | 843.09 | 3.51 | 86.84 | 33.90 | 308.31 |
| **11** | 514.76 | 862.06 | 3.89 | 91.43 | 35.96 | 324.76 |
| **12** | 526.63 | 886.95 | 4.16 | 91.67 | 35.87 | 369.21 |
| **13** | 527.92 | 886.13 | 3.84 | 94.25 | 36.66 | 304.35 |
| **14** | 541.38 | 911.18 | 3.12 | 93.09 | 36.51 | 320.34 |
| **15** | 510.02 | 856.65 | 3.89 | 91.43 | 35.97 | 324.76 |
| **16** | 436.69 | 708.77 | 2.37 | 70.18 | 26.77 | 240.26 |
| **17** | 461.52 | 751.73 | 2.88 | 74.98 | 28.69 | 274.70 |
| **18** | 430.92 | 693.53 | 1.05 | 68.02 | 26.06 | 241.25 |
| **19** | 451.70 | 731.19 | 1.57 | 72.82 | 27.99 | 275.69 |
| **20** | 465.56 | 758.36 | 1.85 | 76.64 | 28.68 | 320.14 |

(*) Numbering as in Table 1.

**TABLE 3**
**Regression Coefficients of One-Variable Equations**

| Descriptor | r(linear) | r(quadratic) | r(cubic) |
|---|---|---|---|
| **V** | 0.9558 | 0.9558 | 0.9561 |
| **S** | 0.9631 | 0.9631 | 0.9635 |
| **Log p** | 0.7031 | 0.7032 | 0.7048 |
| **RM** | 0.9543 | 0.9567 | 0.9568 |
| **P** | 0.9451 | 0.9494 | 0.9507 |
| **M** | 0.8952 | 0.9319 | 0.9319 |

Once again we verify that one can get quite satisfactory results by resorting to higher order polynomials, so that a valid procedure to take full advantage of fitting equations can be made.

The low-average absolute deviations from experimental values derived from the above predictions are particularly notorious. In fact, they represent around 2 and 1.2%, respectively, of the average experimental RIs, which are rather small overall variations. Besides, it is interesting to point out that there are not "deviant" behaviors within the chosen molecular set, so that the present molecular descriptors seem to be suitable for the present purposes of predicting RIs.

Finally, we present a correlation matrix of collinearity of the chosen molecular descriptors in Table 5.

**TABLE 4**
**Experimental and Theoretical Results for RIs/1000**

| Molecule(*) | RI(exp)/1000 | RI/1000 Eq. 1 | RI/1000 Eq. 5 | RI/1000 Eq. 6 |
|---|---|---|---|---|
| **1** | 2.5610 | 2.5956 | 2.5243 | 2.5532 |
| **2** | 2.6389 | 2.6956 | 2.6753 | 2.7043 |
| **3** | 2.8824 | 2.8280 | 2.8710 | 2.8851 |
| **4** | 3.0061 | 3.0659 | 3.0320 | 3.0272 |
| **5** | 2.8285 | 2.8413 | 2.8756 | 2.8496 |
| **6** | 3.0111 | 2.9851 | 2.9681 | 2.9654 |
| **7** | 3.1718 | 3.1993 | 3.2047 | 3.1960 |
| **8** | 2.8605 | 2.8670 | 3.9134 | 2.9167 |
| **9** | 2.5502 | 2.6572 | 2.5653 | 2.5487 |
| **10** | 2.7341 | 2.7529 | 2.7221 | 2.7183 |
| **11** | 2.9838 | 2.8786 | 2.9273 | 2.9287 |
| **12** | 2.9292 | 2.9020 | 2.9638 | 2.9448 |
| **13** | 3.1101 | 3.1071 | 3.0935 | 3.0936 |
| **14** | 3.1072 | 3.0476 | 3.0828 | 3.1110 |
| **15** | 2.9612 | 2.8458 | 2.8897 | 2.8980 |
| **16** | 2.0291 | 2.0610 | 2.0055 | 2.0148 |
| **17** | 2.2679 | 2.3460 | 2.3150 | 2.2809 |
| **18** | 2.1133 | 2.0245 | 2.1153 | 2.1180 |
| **19** | 2.3375 | 2.2815 | 2.3305 | 2.3381 |
| **20** | 2.5456 | 2.5238 | 2.5538 | 2.5406 |
| **Average Absolute Deviation(**)** | - | 0.0496 | 0.0302 | 0.0227 |

(*) Numbering as in Table 1.

(**) $\sum_{i=1}^{n=20} |RI(experimental)_i - RI(theoretical)_i| / n$

**TABLE 5**
**Correlation Matrix of the Chosen Molecular Descriptors**

| r | SAG | V | log p | RM | α | M |
|---|---|---|---|---|---|---|
| **SAG** | 1.00 | 0.99 | 0.74 | 0.97 | 0.97 | 0.81 |
| **V** | 0.99 | 1.00 | 0.75 | 0.98 | 0.98 | 0.80 |
| **log p** | 0.74 | 0.75 | 1.00 | 0.81 | 0.81 | 0.66 |
| **RM** | 0.97 | 0.98 | 0.81 | 1.00 | 0.99 | 0.83 |
| **α** | 0.97 | 0.98 | 0.81 | 0.99 | 1.00 | 0.81 |
| **M** | 0.81 | 0.80 | 0.66 | 0.83 | 0.81 | 1.00 |

## CONCLUSIONS

One of the widely used data reduction techniques, multiple regression analysis, often gives valuable insights into structure-property relationships. However, most often a direct interpretation of the results emerging from such analysis is rather difficult. It is generally understood that QSRR correlations at best suggest a parallel between the quantities involved (evaluators and responses) and do not necessarily reflect a cause-effect relationship[61]. The physical chemistry property studied in this work via the aforesaid quantum-mechanical descriptors—M, SAG, V, log p, RM, and α—are dependent upon the structure in general terms and also are dependent on more subtle quantities, some of which are directly related to these descriptors. This study is a first attempt to thoroughly analyze the influence of the intimate molecular structure on the RIs.

We have presented the results of determining experimental GC and theoretical RIs for a set of 20 1-(2-hydroxyphenyl)-3-arylpropane-1,3-diones. The calculations were made within the frame of the QSRR theory, resorting to six molecular descriptors closely associated with the molecular structure. The resulting fitting equations are quite satisfactory when one appeals to several variable polynomials and takes recourse of computing higher-order formulae. These results are in line with others previously determined for a set of organic bromo and nitrile derivatives, which seems to point out the suitability of the present molecular descriptors for computing RIs.

## ACKNOWLEDGMENT

The authors would like to thank the referee for comments, which have helped to improve the final version of this paper.

## References

1. Ellis, G.P. (1977) In *Chromenes, Chromanones and Chromones. The Chemistry of Heterocyclic Compounds*; Vol. 31.Weissberger, A. and Taylor, E.C., Eds. John Wiley & Sons, New York.
2. Buckler, R.T., Garling, O.L., and Ward, F.E. (1980) U.S., US 4,241,069, Appl. 1979, 72,105; *Chem. Abstr.* 1981, **94**, 139625s.
3. Vibhute, Y.B. and Wadje, S.S. (1976) *Indian J. Exp. Biol*. **14**, 739; *Chem. Abstr.* **1977**, 86, 38005u.
4. Vibhute, Y.B. (1976) *J. Indian Chem. Soc*. **53**, 736.
5. Subbanwad, G.R. and Vibhute, Y.B. (1992) *J. Indian Chem. Soc*. **69**, 337; *Chem. Abstr.* **1993**, 118, 212714r.
6. Attasi, G., Briet, P., Berthelon, J.J., and Collonges, F. (1985) *Eur. J. Med. Chem. Chim. Ther.* **20**, 393; *Chem. Abstr.* **1986**, 104, 122574b.
7. Briet, P., Berthelon, J.J., and Collonges, F. (1984) Fr. Demande, FR 2,536,397, Appl. 1982, 82/19,640; *Chem. Abstr.* **1984**, 101, 230357e.
8. Emsley, J. (1984) *Struct. Bonding* **57**, 147
9. Rappoport, Z., Ed. (1990) *The Chemistry of Enols.* John Wiley & Sons, New York.
10. Cohen, B. and Weiss, S. (1984) *J. Phys. Chem.* **88**, 3159.
11. Lazaar, K.I. and Bauer, S.H. (1984) *J. Phys. Chem.* **87**, 2411.
12. Cravero, R.M., González-Sierra, M., and Olivieri, A.C. (1993) *J. Chem. Soc. Perkin Trans*. **2**, 1067.
13. Geraldes, C.F.G.C., Barros, M.T., Maycock, C.D., and Silva, M.I. (1990) *J. Mol. Struct*. **238**, 335.
14. Gorodetsky, M., Luz, Z., and Mazur, Y. (1967) *J. Am. Chem. Soc*. **89**, 1183.
15. Bertolasi, V., Gilli, P., Ferreti, V., and Gilli, G. (1991) *J. Am. Chem. Soc.* **113**, 4917.
16. Hibbert, F. and Emsley, J. (1990) *Adv. Phys. Org. Chem*. **26**, 255.
17. Grens, E., Grinvalde, A., and Stradins, J. (1990) *Spectrochim. Acta* **31A**, 555.
18. Fujii, H., Yoshimura, T., and Kamada, H. (1997) *Tetrahedron Lett*. **38**, 1427.
19. Bouchoux, G., Hoppilliard, Y., and Houriet, R. (1987) *Nouv. J. Chim*. **11**, 225.
20. Peake, D.A., Stanley, J., Kingsbury, C.A., and Gross, M.L. (1986) *Org. Mass Spectrom*. **21**, 565.
21. Masur, M., Grützmacher, H.F., Münster, H., and Budzikiewicz, H. (1987) *Org. Mass Spectrom*. **22**, 493.
22. For a detailed review of crystalline structure determinations up to 1988 see reference 9: Gilli, G. and Bertolasi, V., Chapter 13 and references cited herein.
23. Ramos, M., Alkorta, I., and Elgero, J. (1997) *Tetrahedron* **53**, 1403.
24. Karelson, M., Maran, U., and Katritzky, A.R. (1996) *Tetrahedron* **52**, 11325.
25. Jios, J.L., Autino, J.C., and Pomilio, A.B. (1995) *An. Asoc. Quim. Argent*. **83**, 183.
26. Jios, J.L. (1996) Doctoral Thesis. Universidad de La Plata, Argentina.
27. Jios, J.L. and Duddeck, H. (2000) *Z. Naturforsch*. **55b**, 193.
28. Jios, J.L. and Duddeck, H. (2000) *Magn. Res. Chem*. **38**, 512.
29. Echeverría, G., Jios, J.L., Autino, J.C., and Punte, G. (2000) *Structur. Chem*. **6**, 367.
30. Osmialowski, K., Halkiewicz, J., Radecki, A., and Kaliszan, R. (1985) *J. Chromatogr*. **346**, 53.
31. Buydens, L., Georgakopoulos, C.G., Kiburis, J.C., and Jurs, P. (1991) *Anal. Chem*. **63**, 2021.
32. Bermejo, J. and Guillen, M. (1987) *Anal. Chem*. **59**, 94.
33. Woloszyn, T. and Jurs, P. (1992) *Anal. Chem*. **64**, 3059.
34. Katritzky, A., Ignatchenko, E., Barcock, R., Lobanov, V., and Karelson, M. (1994) *Anal. Chem*. **66**, 1799.
35. Kováts, E. (1961) *Anal. Chem*. **181**, 351.

36. Kaliszan, R. (1987) *Quantitative Structure-Chromatographic Retention Relationships*. John Wiley & Sons, New York.
37. Zenpurich, I.G. (1999) *Fresenius J. Anal. Chem*. **365**, 305.
38. McLafferty and Stanffer, D.B. (1991) *Important Peak Index of the Registry of Mass Spectral Data*. John Wiley & Sons, New York.
39. Kaliszan, R. (1989) In *High Performance Liquid Chromatography*. Brown, P.R. and Hartwick, R.A., Eds. John Wiley & Sons, New York.
40. Kaliszan, R. (1993) *J. Chromatogr*. **656**, 417.
41. Kaliszan, R., Markuszewski, M., Haber, P., Nasal, A., Cserháti, T., Forgács, E., Gadzala-Kopciuch, R.M., and Buszewski, B. (1998) *Chem. Anal. (Warsaw)* **43**, 547.
42. Cochi, M., Menziani, M.C., De Benedetti, P.G., and Cruciani, G. (1992) *Chemom. Intell. Lab. Sysy.* **14**, 209.
43. Katritzky, A.R., Petrukhin, R., and Tatham, D. (2001) *J. Chem. Inf. Comput. Sci.* **41**, 679 and references therein.
44. Viswanadhan, V.N., Ghose, A.K., Revankar, G.N., and Robins, R.K. (1989) *J. Chem. Inf. Comput. Sci*. **29**, 163.
45. Ghose, K. and Crippen, G.M. (1987) *J. Chem. Inf. Comput. Sci*. **27**, 21.
46. Bodor, N., Gabanyi, Z., and Wong, C.J. (1989) *Am. Chem. Soc*. **111**, 3783.
47. Gavezotti, A. (1983) *J. Am. Chem. Soc*. **100**, 5220.
48. Miller, K.J. (1990) *J. Am. Chem. Soc*. **112**, 8533.
49. Chem Plus Extension for Hyperchem® Modelling for Windows™. (1994) Hypercube, Inc., Gainesville, Florida.
50. Wolfram, S. (1999) *The Mathematica® Book: Fourth Edition*. Wolfram Media, Cambridge University Press, Cambridge.
51. Tan, P.T. (1997) *A Physicist's Guide to Mathematica®*. Academic Press, New York.
52. Duchowicz, P. and Castro, E.A. (2000) *J. Korean Chem. Soc*. **44**, 501.
53. Romanelli, G.P., Cafferata, L.F.R., and Castro, E.A. (2000) *Khim. Fiz.* **19**, 105.
54. Duchowicz, P. and Castro, E.A. (2000) *Acta Chem. Slov*. **47**, 281.
55. Firpo, M., Gavernet, L., and Castro, E.A. (1999) *Rom. J. Phys*. **44**, 181.
56. Castro, E.A. (1997) *Comp. Chem*. **21**, 305.
57. Livingstone, D. (1995) *Data Analysis for Chemists.* Oxford Science Publications, Oxford University Press, Oxford. pp.127–129.
58. Hansch, C. (1969) *Acc. Chem. Res*. **2**, 232.
59. Hansch, C., Steward, A.R., Anderson, S.M., and Bentey, J. (1968) *J. Med. Chem*. **11**, 1.
60. van de Waterbeemd, H. and Kansy, M. (1992) *Chimia* **46**, 299.
61. Randic, M. (1991) *J. Mol. Struct. THEOCHEM* **232**, 45.
